# Validity and Reliability of the Persian Version of Leeds Dyspepsia Questionnaire

**DOI:** 10.31661/gmj.v8i0.1609

**Published:** 2019-12-31

**Authors:** Sepideh Batebi, Abbas Masjedi Arani, Mahdi Jafari, Amir Sadeghi, Mohsen Saberi Isfeedvajani, Mohammad Hassan Davazdah Emami

**Affiliations:** ^1^Department of Clinical Psychology, Faculty of Medicine, Shahid Beheshti University of Medical Sciences, Tehran, Iran; ^2^Gastroenterology and Liver Diseases Research Center, Research Institute for Gastroenterology and Liver Diseases, Shahid Beheshti University of Medical Sciences, Tehran, Iran; ^3^Medicine, Quran and Hadith Research Center & Department of Community Medicine, Faculty of Medicine, Baqiyatallah University of Medical Sciences, Tehran, Iran; ^4^Department of Health Psychology, Isfahan University of Medical Science, Isfahan, Iran

**Keywords:** Dyspepsia, Surveys and Questionnaires, Reproducibility of Results, Gastrointestinal Diseases, ROC Curve, Sensitivity and Specificity, Gastroesophageal Reflux

## Abstract

**Background:**

It is essential in clinical care services to measure the symptoms of functional dyspepsia both in the primary examination and treatment outcomes. No valid assessment tool is already available for functional dyspepsia in Iran. The present study aimed at evaluating the reliability, validity, and responsiveness of the Leeds dyspepsia questionnaire (LDQ).

**Materials and Methods:**

The LDQ was completed by 67 subjects with no dyspepsia symptoms and 93 subjects with certain functional dyspepsia diagnosed via endoscopy by a gastroenterologist and other clinical assessments. After definite diagnosis of functional dyspepsia, the participants were assessed by the LDQ. The psychometric characteristics of the questionnaire were then documented to investigate its reliability, validity, and responsiveness.

**Results:**

The internal consistency of the LDQ ranged from 0.80 to 0.89 and its test-retest reproducibility was 0.96. The LDQ was significantly correlated with all domains of dyspepsia symptom severity index (DSSI) and also with some of the domains of gastrointestinal symptom rating scale (GSRS). The LDQ had a sensitivity of 90.3% with a great specificity and a very good predictive validity. Moreover, a significant responsiveness to changes was observed (P<0.05).

**Conclusion:**

The LDQ is a valid, reliable, reproducible, and self-rated instrument responsive to change, which can be used to measure the frequency and severity of functional dyspepsia symptoms in clinical trials.

## Introduction

Dyspepsia is a common disorder with the typical upper gastrointestinal symptoms. It affects approximately 2.9%-29% of the Iranian adult population and also is one of the most common causes for referral to gastrointestinal healthcare services [1-5]. According to studies, approximately 30%-50% of peptic ulcer, cholelithiasis, reflux disease, and malignancy cases have organic causes; however, routine clinical diagnoses disclosed no organic causes for functional dyspeptic symptoms thus far [6]. Patients who do not have any organic causes for their dyspepsia symptoms are referred to as functional or non-ulcer dyspeptic cases. Patients with dyspepsia have no problem with their daily living activities, although the disease symptoms affect their quality of life and impose a substantial economic burden on the health systems and society [7-12]. Although dyspepsia has widely been investigated, its management is still unknown [13]. The symptoms of functional dyspepsia include epigastric pain, retrosternal pain, regurgitation, nausea, vomiting, belching, bloating, and dysphagia [14, 15]. However, providing a definition for the symptoms and an effective multidimensional scale to measure generalizability, frequency, and severity of the symptoms affecting patients’ quality of life is questionable [16-18]. An appropriate questionnaire is needed to precisely assess the frequency and severity of functional dyspepsia, considering both physician’s diagnosis and endoscopic results through various phases of treatments; hence, some strategies should be developed for initial assessments. Furthermore, in the gastrointestinal healthcare services of Iran, no valid questionnaire for the assessment of dyspepsia is used. A well-validated questionnaire should precisely differentiate patients with dyspepsia from those without it and consider the symptoms severity and follow-ups. The current study aimed at validating the revised full version of the Leeds dyspepsia questionnaire (LDQ) for the initial evaluation and assessment of change in treatment process.

## Materials and Methods

### Translation of the questionnaire

First, the forward-translation method was used to translate the standard English copy of the LDQ into Persian by a qualified translator and then trained clinical specialists reviewed the translated version in order to check its accuracy in terms of concept and semantics. Next, the translated version was back-translated into English by an experienced English translator. Finally, after being assured of the congruence between the Persian and English versions, the study was conducted. After obtaining a general consensus on the forward-translated Persian version of the LDQ, a pilot study with 10 subjects of different ages and various socio-economic levels was conducted and a consensus was achieved among the clinicians in order to make the items conceptually and semantically understandable.

### Evaluation of the questionnaire

The LDQ contained eight main questions about dyspeptic symptoms (epigastric pain, retrosternal pain, dysphagia, regurgitation, belching, nausea, vomiting, bloating) categorized according to frequency and severity. The first question examined the presence of dyspeptic symptoms followed by those measuring frequency and severity. In addition, the questionnaire included an extra question about troublesome symptoms during the last month, which was not added to the total score for better differentiation of epigastric and heartburn symptoms. The full version of the LDQ included items on the frequency of symptoms; frequency items were scored from 0 (never) to 5 (most often) and the severity ones from 0 (not at all) to 5 (very severe). Higher scores represented more severe and lower scores indicated less severe functional dyspepsia symptoms, which affected patients’ daily living activities, such as eating, sleeping, working, and leisure time over the last month. A diagram was used for two main questions assessing epigastric and retrosternal pain in order to distinguish them more easily.

Following endoscopic investigations, the patients were examined by a gastroenterologist for dyspeptic symptoms according to the Rome IV criteria. In addition, as a quasi-gold standard test to validate the questionnaire, the definitive diagnosis of an experienced gastroenterologist after endoscopic investigation of the target patients was received. The patients were asked to participate in the study and the informed consent was obtained from them. The LDQ was completed by 67 subjects with no dyspepsia symptoms and 93 subjects with certain functional dyspepsia diagnosed via endoscopy by a gastroenterologist and other clinical assessments. The inclusion criteria were: age range from 18 to 65 years, lack of non-organic or functional symptoms of dyspepsia in the subjects without dyspepsia, lack of organic causes of dyspepsia and comorbidity of diseases such as irritable bowel syndrome (IBS) with functional dyspepsia in the subjects with functional dyspepsia, lack of chronic somatic diseases, lack of simultaneous use of psychiatric drugs, and lack of substance abuse or dependence. The exclusion criteria were: unwillingness to participate in the study for any reason, lack of simultaneous use of medications during the study period, and lack of participation to other therapeutic assessments until the reevaluation phase. A total of 160 participants, including 93 subjects with functional dyspeptic and 67 subjects without dyspepsia symptoms, were enrolled in initial evaluations; after an interval two weeks, 90 subjects, including 50 patients with functional dyspeptic and 40 subjects without dyspepsia symptoms were entered for the test-retest reproducibility. Forty-two patients were diagnosed with functional dyspepsia via endoscopic and clinical examinations that were subjected to treatment with nortriptyline 25 mg, omeprazole 25 mg, and domperidone 25 mg (once a day) with regular physical activity and a healthy diet for two months in order to investigate the responsiveness of the LDQ to change under the common treatment. Two other questionnaires, including dyspepsia symptom severity index (DSSI) and gastrointestinal symptom rating scale (GSRS) were also utilized to assess gastrointestinal symptoms in order to explore the congruent and discriminant validity of the LDQ. DSSI is a 20-item self-report questionnaire addressing dyspepsia and reflux symptoms. The items are scored based on a five-point Likert scale from 0 (absent) to 4 (very severe); DSSI has three subscales as ulcer-like, dysmotility-like, and reflux-like dyspepsia. Total score is obtained by the mean scores across subscales. Cronbach’s alpha coefficient was 0.76-0.80 and accordingly the internal consistency of the subscales was high (0.84-0.89) [19]. In the current study, DSSI and LDQ were filled out by 80 subjects, including 50 patients with and 30 subjects without functional dyspepsia in order to measure the congruent validity. Likewise, to study the discriminant validity, the GSRS assessing general gastrointestinal disorders, i.e., IBS was used. The GSRS is a 15-item disease-specific instrument containing five subscales as abdominal pain (abdominal pain, hunger pain, and nausea), indigestion (borborygmus, abdominal distention, eructation, and excessive flatus), gastrointestinal reflux (heartburn and acid reflux), diarrhea (diarrhea, loose stools, and urgent need for defecation), and constipation (constipation, hard stools, and a sensation of incomplete evacuation). The items are scored based on a seven-point Likert scale from 0 (the absence of troublesome symptoms) to 7 (very troublesome symptoms). The total score of the GSRS is the mean scores across subscales. A higher score represents more severe gastrointestinal disorder. The GSRS represented acceptable internal consistency reliability, responsiveness to change, and reasonable construct validity among the European and Iranian populations. Accordingly, it is considered as a useful patient-rated scale to evaluate the gastrointestinal symptoms after treatment [20, 21]. The validity and reliability of the GSRS for both patients and general population are well documented. its internal consistency reliability ranged from 0.43 to 0.87 [22]. In addition, the internal consistency reliability of the validated Persian version of GSRS for all its domains in patients with functional gastrointestinal disorders and healthy subjects were 0.63-0.80 and 0.36 - 0.80, respectively [21]. In the current study, 60 subjects with and 30 ones without functional dyspepsia completed the GSRS in addition to the LDQ. The internal consistency, test-retest reliability, concurrent and discriminant validity, sensitivity and specificity, and positive and negative predictive values were tested using IBM SPSS Statistics for Windows, version 19 (IBM Corp., Armonk, N.Y., USA).

## Results

### Study Population

A total of 160 participants, 93 subjects with functional dyspepsia and 67 subjects without dyspepsia symptoms were studied, of which 70% were female and 30% male within the age range of 18 to 65 years and the mean age of 35 years. The majority of the participants were high school graduates (35.6%); in addition, 34.4% were single, and 65.6% married. Seventeen of the subjects with no dyspepsia symptoms were excluded. Therefore, the statistical analysis was performed on 143 subjects.

### Internal consistency

According to Table-1, Cronbach’s alpha was 0.92, representing a high level of internal consistency among the scale items. Cronbach’s alpha for each item ranged from 0.80 to 0.89, which means that each item has an appropriate correlation with the entire questionnaire.

### Test-retest reliability

Of 90 participants, 50 subjects with and 40 ones without functional dyspepsia were entered in the retest phase using the LDQ after two weeks. Pearson’s correlation coefficient between the first and second total scores was 0.96 (P<0.05) and ranged from 0.82 to 0.92, indicating a high level of test-retest reliability.

### Construct validity

Concurrent validity was assessed by comparing the LDQ total score and symptoms subscales with the total score and subscales of the DSSI. The correlation between the two total scores was 0.93. The total score correlation of the LDQ with dysmotility-like dyspepsia was 0.92, with reflux-like dyspepsia was 0.84, and with ulcer-like dyspepsia was 0.82. In terms of subscales, the correlation of dysmotility-like dyspepsia with bloating was 0.85, with epigastric pain was 0.84, and with nausea was 0.73; the correlation of reflux-like dyspepsia with regurgitation was 0.81 and with epigastric pain was 0.74; ulcer-like dyspepsia was highly correlated with epigastric pain (0.90; P<0.05), suggesting a good concurrent validity.

### Discriminant validity

Discriminant validity was assessed by comparing the correlation between the LDQ subscales and GSRS. The GSRS is designed to evaluate common gastrointestinal disorders and IBS symptoms. It was found that some of the GSRS domains were correlated, whereas other domains (those assessing IBS) had no correlation with the LDQ. Pearson’s correlation coefficient between the LDQ and abdominal pain, nausea, and indigestion were 0.86, 0.82, and 0.86, respectively. There was no correlation between constipation and diarrhea according to the symptoms of IBS (P<0.05).

### Sensitivity analysis

**Figure 1:**
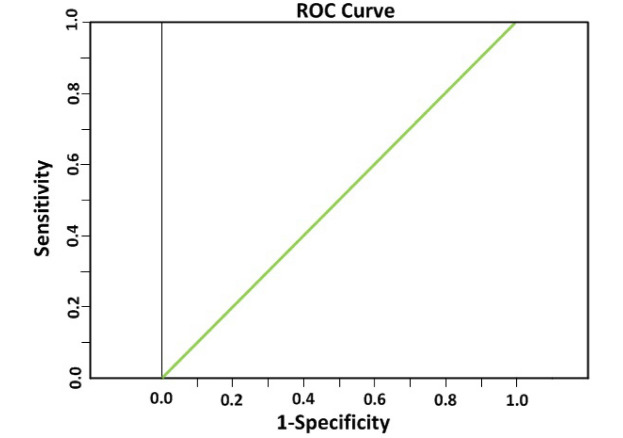
The receiver operating characteristic curve demonstrating the sensitivity and specificity of the Leeds dyspepsia questionnaire to differentiate patients with functional dyspepsia from the ones without it.

For the total score of LDQ, the characteristics of receiver operating characteristic (ROC) curve are shown in Figure-1 to demonstrate the sensitivity and specificity of the questionnaire. According to Table-2, the area under the ROC curve of the LDQ total score was 0.99. Accordingly, a large area with a confidence interval of 95% was statistically significant. The best cutoff point to diagnose functional dyspepsia was 16.5, where sensitivity was 90.3% with a great specificity of about 100%, which resulted in a considerable precision to differentiate patients with functional dyspepsia from the ones without it. In addition, the positive and negative predictive values were 96% and 100%, respectively indicating a good sensitivity and specificity.

### Responsiveness to change

A total of 93 patients with functional dyspepsia were treated with nortriptyline 25 mg, omeprazole 25 mg, and domperidone 25 mg (once a day) with regular physical activity and a healthy diet for two months. The subjects were then asked to complete the full version of the LDQ in order to prove the efficiency of the scale in evaluating the symptoms changes following the treatment. Eight patients out of 50 patients were excluded, three patients due to the lack of access to proper treatment and five others because of lack of access to follow-up. Accordingly, the final analysis was conducted on 42 patients (84%), who filled out the second full version of the questionnaire following the treatment. The mean total score fell from 21.33± 4.52 to 19.26 ± 4.25, suggesting the responsiveness of the LDQ to change following treatment ( Table-3; P<0.05).

## Discussion

The study evaluated the psychometric validity and reliability of the Persian version of the LDQ and the results showed that it is the most reliable and valid instrument with a high responsiveness to assess the frequency and severity of functional dyspepsia symptoms. The studied LDQ was a valid patient-rated scale, since it had high sensitivity and specificity in the diagnosis of functional dyspepsia. The results were consistent with those of previous studies reporting a sensitivity of 70.0% and 73.1% and specificity of 71.2% and 66.6% for two different versions of the LDQ Malaysian-English and Malay, respectively [15]. In a study conducted on general population, the sensitivity and specificity of the LDQ were respectively 80% and 79%, whereas they were 99% and 53%, respectively in hospitalized patients [1]. The reported cutoff point in the present study was ≥16.5, which is consistent with a recent study [23]. In the study, the LDQ had a high predictability to differentiate between the patients with functional dyspeptic and the ones without it, which can be attributed to good positive and negative predictive values of the instrument. The LDQ had a good responsiveness to change and demonstrated an adequate construct validity included concurrent and discriminant validity. The LDQ was found as a reliable instrument to measure the changes in responsiveness to the treatments, so it can be used to make comparisons over time, which is consistent with the results of previous studies [1, 15]. In terms of psychometric characteristics, its internal consistency reliability for the total score and subscales was desirable. The internal consistency of the subscales was high, which is consistent with the results of previous studies reporting an excellent internal consistency for the questionnaire [1, 15, 23]. The LDQ test-retest reliability was significantly acceptable for all subscales. The highest correlation was observed in the regurgitation and the lowest in nausea, confirming the validity of the measurement tool. In accordance with recent studies, its test-retest reliability for the Malay and Malaysian-English versions were 0.71 and 0.77, respectively [15]; the test-retest reliability was 0.83 for the English version [1], and 0.89 for the Chinese version [23]. These results represent the excellent reproducibility of the studied LDQ. Moreover, the missing data resulted from unanswered questions were negligible indicating that the scale was acceptable and understandable for the participants. Future studies may compare the accuracy of diagnosis by endoscopy and the LDQ the detection of functional dyspepsia before and after treatments. Further investigations are required to assess the frequency and severity of functional dyspepsia through comparing the reports provided by patients and physicians. In future studies on functional dyspepsia symptoms, the differences and similarities of the results obtained from the Persian version of the LDQ should be compared with the gastroenterologist’s diagnosis in the early and secondary evaluation stages after treatment. To the best of authors’ knowledge, there was no valid Persian version of the LDQ in the clinical population, general population, and clinical trials. This questionnaire can also be reliably used in studies on the general population to detect dyspepsia. All in all, the LDQ showed a good internal consistency and reliability, it was excellent in precise diagnosis and demonstrated a good test-retest reliability.

## Conclusion

It can be concluded that all domains of the LDQ are reliable with an appropriate internal consistency and responsiveness in evaluating the frequency and severity of the symptoms. The results of the present study indicated that the LDQ can be used in clinical practice.

## Acknowledgement

The authors wish to express their gratitude to their colleagues in the Research Institute for Gastroenterology and Liver Diseases of Shahid Beheshti University of Medical Sciences, Tehran, Iran, for providing insights and expertise that greatly helped them to conduct the research. The authors are immensely grateful to Drs. Mohammad Reza Zali, Majid Iranshahi, and Saeed Abdi for their assistance as well as Dr. Mohammad Rostami Nejad for his comments that greatly improved the manuscript.

## Conflict of Interest

The authors declare no conflict of interest.

**Table 1:** Reliability Statistics for the Persian Version of LDQ

**Questionnaire**	**Items**	**Cronbach’s Alpha**
LDQ	16	0.924

*P<0.05

**Table 2:** Area Under the ROC Curve with a Confidence Interval of 95%

**Area**	**Std. Error** ^+^	**Asymptotic 95% Confidence Interval**	
		**Asymptotic Sig.** ^++^	**Lower Bound**	**Upper Bound**
0.993	0.004	0.000	0.986	1.000

^+^: under the nonparametric assumption ^++^: Null hypothesis, true area: 0.05 *P<0.05

**Table 3:** Paired Comparison of the LDQ Score before and after the Treatment

**Mean**	**Std.** **Deviation**	**Std.Error** **Mean**	**T**	**Df**	**Sig.** **(2- tailed)**
2.071	3.241	0.500	4.141	41	0.000

*P<0.005
